# Much room for change: access to surgical care for stateless individuals in Pakistan

**DOI:** 10.1186/s12992-023-00972-3

**Published:** 2023-11-28

**Authors:** Humza Thobani, Mashal Murad Shah, Anam N Ehsan, Sadaf Khan

**Affiliations:** 1 Department of Surgery, Aga Khan University, Karachi, Pakistan; 2 Centre of Global Surgical Care, Aga Khan University, Karachi, Pakistan; 3 Brigham and Women’s Hospital, Boston, MA USA

**Keywords:** Statelessness, Global surgery, Health service access

## Abstract

**Background:**

As developing countries take steps towards providing universal essential surgery, ensuring the equitable distribution of such care for underrepresented populations is a vital function of the global surgery community. Unfortunately, in the context of the global “stateless”, there remains much room for improvement.

**Key issues:**

Inherent structural deficiencies, such as lack of adequate population data on stateless communities, absent health coverage policies for stateless individuals, and minimal patient-reported qualitative data on barriers to surgical service delivery prevent stateless individuals from receiving the care they require – even when healthcare infrastructure to provide such care exists. The authors therefore propose more research and targeted interventions to address the systemic issues that prevent stateless individuals from accessing surgical care.

**Conclusion:**

It is essential to address the aforementioned barriers in order to improve stateless populations’ access to surgical care. Rigorous empirical and qualitative research provides an important avenue through which these structural issues may be addressed.

## Background

The exodus of the Rohingya community from Myanmar over the past several years and their subsequent citizenship crisis has drawn much global attention and scrutiny towards the issue of statelessness [1]. Defined by the World Health Organization (WHO) as individuals who are “not recognized as a national by any state under the operation of its law”, statelessness is a unique legal identity with an equally unique set of public health challenges [2].

Stateless individuals share numerous key similarities with refugees, and therefore they are both awarded the same asylum status and protections by the International Convention on the Reduction of Statelessness and International Bill of Rights [3]. However, the primary difference between the two groups stems from the fact that refugees have some form of legal or documented proof of citizenship of a particular state, whereas stateless individuals do not. Refugees are still citizens of the country they left, while stateless individuals do not hold any form of citizenship or legal belonging to any state. Therefore, while it may be tempting to view this distinction as arbitrary, the legal and health challenges faced by stateless individuals are often separate and dissimilar to those faced by refugees, thereby necessitating focused and targeted study for intervention and health improvement.

This can be contextualized in a practical sense. It is well known that refugees are at high risk for poor health outcomes, as they fare poorly in numerous key social determinants of health, such as lacking income, food security and safety [4, 5]. Stateless individuals are constricted by many of the same factors, with one key difference: they lack any form of meaningful upward social mobility to improve their socioeconomic standing. Without any form of legal identity, stateless people have no legal standing to apply for citizenship, asylum or social welfare [5]. The outcome: millions of stateless individuals (and often their children) have existed in the exact same conditions for years, often with devastating effects on their health.

This is precisely what is seen with the Bengali and Burmese stateless community in Karachi, Pakistan. Despite living in settlements within minutes from some of the city’s largest public hospitals tens of thousands of stateless residents have never seen the inside of a hospital clinic [6]. Born to ethnic Bengalis who settled in Pakistan decades prior in the early 1970s, they are not recognized as citizens of either Pakistan or Bangladesh. Although accurate census data is difficult to attain, a 2020 Global Appeal Report by the Office of the UN High Commissioner for Refugees (UNHCR) estimates that there are hundreds of thousands of such “stateless” individuals residing in Pakistan [7].

Yet, despite being fully assimilated into Pakistani culture, they are unable to access basic state and non-state services, such as education, healthcare, or even the ability to buy and own property [8, 9]. As a result, stateless individuals are highly marginalized and forced by necessity to live in informal urban settlements on daily wage incomes [8]. Ironically, many fulfil the criteria for citizenship set by the Pakistan Citizenship Act 1951 but are unable to apply for it due to difficulties navigating the highly convoluted, inefficient, and at times discriminatory bureaucratic registration process [10, 11]. It is important to note that in the near absence of existing literature on these stateless individuals, these observations are made and based primarily on expert editorials and investigative journalism cover pieces [6, 12].

With modern globalization and increased inter-connectivity between states, it is likely that the global population who are stateless and their health needs will continue to grow [13]. As a result, increasing attention has been given to the public health interests of stateless persons in recent literature [14–16]. However, we argue that as researchers attempt to uncover the health challenges faced by stateless persons and communities, it is crucial not to exclude surgical care from the overall paradigm of healthcare provision. We contend that the treatment of diseases requiring surgery is an essential component of health and the unmet need for surgical care must be considered in future healthcare provision for stateless communities. In addition, there needs to be a better understanding of the issues faced by stateless individuals in accessing surgical care such that appropriate interventions can be implemented.

## Main body

In the context of universal health coverage and essential surgery, current research tends to be centered around two central themes of importance: cost and access [17]. For the latter, global surgery literature often focuses on health service delivery in rural areas where surgical capacity is severely limited or non-existent [18–20]. The stateless individual therefore presents a paradox in surgical health policy. Many statelesspeople in Pakistan reside in urban centers such as Karachi with established healthcare and surgical infrastructure which is well within a two hour commute distance [6]. Yet, as they are ineligible to register as citizens and are simultaneously not afforded the protections and legal status of a refugee, they cannot access care despite it being available [21]. Further, public hospitals often provide surgical care which is either free of cost or heavily subsidized, meaning that if identification is provided, the direct medical costs of care are often less of a hindrance.

Statelessness is a highly complex legal issue with massive variability in protections for displaced persons between states [22]. Therefore, it would be imprudent to attempt to identify a one-size-fits-all solution for the lack of surgical access for stateless individuals. Country-specific lobbying and political action to award more legal protections for such populations would likely yield the greatest results – a feat very much outside the scope of this paper. In the interim, the authors contend that the role of a global health researcher would be to facilitate this process by identifying structural deficiencies in current health systems through meaningful empirical research. The authors therefore outline the following thematic areas for future study, identify potential structural barriers to access to surgical care for stateless individuals and propose directions for future research to help overcome these barriers.

### Census data for stateless individuals

A large part of the problem is a stark lack of epidemiologic data necessary to undertake appropriate interventions. Stateless populations are often not included in healthcare planning and census data, making it nearly impossible to estimate the scale of healthcare needs of this group. The 2017 Pakistan Population and Housing Census specifically references “Afghan refugees” as a category in its final report, but makes no mention of stateless individuals [23]. It is not immediately clear as to why stateless individuals have been excluded from census. In addition, the report also specifies that refugees living in “Refugee Villages” were not counted in the census while those residing in rural and urban centers were. Again, no explanation is provided for this sampling strategy and thus, serves to make census statistics on refugees and stateless individuals ambiguous and less reliable.

This problem is by no means limited to Pakistan, and there is also extremely sparse global health literature attempting to quantify the scale of statelessness or the unmet need for surgical care internationally. Most studies evaluating access to surgical care do not clearly distinguish between stateless persons and other legal identities, often resorting to non-specific terms such as “asylum seekers” or “displaced persons” [24–26]. Cavalcante and colleagues were able to identify a small cohort of stateless persons in Rio de Janeiro through a review of asylum registration applications [27]. However, this indirect census method is unlikely to be effective in Pakistan where stateless persons are either unable to register with their host state, or are unwilling to do so due to fear of persecution if they are identified.

Such unreliable data are not appropriate nor sufficient in matters of health policy and planning, especially for relatively more costly (yet cost-effective) health services such as surgical care, where accurate census data help guide the location and scale of interventions. Without even knowing how many stateless individuals reside in a particular area at a particular time, it is nearly impossible to attempt to identify ways to improve their access to surgical care or to fully comprehend the unmet needs.

Global health research is therefore crucial to quantify what exactly the unmet need for surgical care is. Public policy relies heavily on population data to accurately model health investment and expenditure [28]. Research such as community-based surgical needs assessments targeting stateless persons can help fill a substantial gap in knowledge regarding the scope of the unmet need for surgical care amongst stateless persons. There is precedent for this in current literature related to rural and disadvantaged populations, and we believe similar research for stateless populations would be an essential interim measure to address the problems outlined previously until more accurate census level data becomes available [25, 29, 30].

### Cost considerations and coverage policies for surgical health service delivery

A natural prerequisite for healthcare access is the ability to present some form of personal identification at the first point of contact. Smaller primary or community care initiatives such as outpatient preventative health visits or immunization drives may be able to waive this requirement, but the nature of inpatient, surgical and obstetric care in most healthcare systems often necessitates that some form of identification documentation be provided [31]. This is often tied to an individual’s right to healthcare financing and compensation, as eligibility for government or private coverage requires some form of personal identification as a means to audit and trace payouts to individuals [31, 32].

This is the case in Pakistan, as access to hospital-based care is often restricted to registered citizens – for example, in the Punjab province, one can only apply for a “Sehat Sahulat card” to access healthcare financing if they can provide proof of citizenship [33]. Importantly, this limitation does not apply for primary outpatient care. Community level care is universally accessible in many areas of Pakistan and is delivered door to door by the Lady Health Worker (LHW) program, whereas routine care is provided by various clinics and public-private partnerships [34]. Furthermore, as primary care gatekeeping is not mandatory or universally implemented in Pakistan, the first point of contact for many patients is often a secondary or tertiary care clinic [35]. Therefore, as outpatient payouts are significantly smaller and relatively more affordable, individuals may still access primary care by simply paying out-of-pocket without the need for legal identification [21]. Taking the example of Machar Colony: while small-scale targeted welfare and relief clinics may provide basic community healthcare, obstetric or surgical emergencies go largely unattended. This often leads to devastating consequences as such conditions necessitate visits to a secondary or tertiary health facility [36].

Heath coverage is a complex topic and is strongly influenced by economic interests and feasibility – can Pakistan and other LMICs with limited capital for health expenditure afford to provide coverage for “expensive” surgery to millions of stateless persons residing within their borders? We argue that global health researchers are the best suited to answer that question through economic evaluation and cost-effectiveness research. Extensive research has shown that surgical care is a highly cost-effective intervention as it averts significant morbidity and mortality associated with surgical disease [37]. Empirical research modelling the fiscal impact of providing surgical care to stateless persons provides an economic justification to pursue coverage policies in addition to the moral imperative to do so.

### Patient reported priorities for surgical service delivery

Perhaps the greatest deficiency in current health literature on the topic is the exclusion of stateless individuals from discourse on the unmet need of surgical care within their communities, and in the global surgery community at large. To the best of the authors’ knowledge, minimal literature exists exploring the perspectives of stateless individuals themselves on the barriers they face to access surgical care. However, there is set precedence for qualitative research in stateless communities for mental health and sexual and reproductive health care [38, 39]. This conversation must be expanded to the surgical needs of stateless populations and the obstacles to healthcare, particularly in Pakistan.

Objective, empirical research may be undertaken as suggested earlier in this paper, however it is unlikely to be as effective without subjective data and knowledge on issues identified by the target population themselves. Meaningful qualitative data in this regard can help guide focused research, understand the barriers and guide interventions and policymaking to address these issues.

## Conclusion

As with any disadvantaged or underrepresented group, improving health service delivery and access for stateless individuals is by any means a challenge. Complex ethnic, social, political and historical factors exacerbate the issue further and may cause nation states and the international community at large to drag their feet in remedying the problem, despite their obligation to uphold the rights of stateless individuals. While the global health community works towards providing solutions with regards to primary and preventative care, the authors argue that the rights of the stateless individual to access surgical care are equally essential and should be given due importance. Given the marked heterogeneity in legislative protections for people who are stateless worldwide, contextual and nation-specific research along the themes of improving the quality of census data, analyzing and assessing requirements for identification documentation, and undertaking qualitative research may provide the foundation for meaningful change ahead.

## Data Availability

This comment contains no supplementary data or materials.

## Abbreviations

ASEAN: Association of South East Asian Nations
UNHCR: United Nations High Commission for Refugees
WHO: World Health Organization
